# Inflammation and Oxidative Stress: The Molecular Connectivity between Insulin Resistance, Obesity, and Alzheimer's Disease

**DOI:** 10.1155/2015/105828

**Published:** 2015-11-26

**Authors:** Giuseppe Verdile, Kevin N. Keane, Vinicius F. Cruzat, Sandra Medic, Miheer Sabale, Joanne Rowles, Nadeeja Wijesekara, Ralph N. Martins, Paul E. Fraser, Philip Newsholme

**Affiliations:** ^1^School of Biomedical Sciences, Curtin Health Innovation Research Institute, Biosciences, Curtin University, Kent Street, Bentley, Perth, WA 6102, Australia; ^2^Centre of Excellence for Alzheimer's Disease Research and Care, School of Medical Sciences, Edith Cowan University, 270 Joondalup Drive, Joondalup, Perth, WA 6027, Australia; ^3^Department of Physiology and Biophysics, Institute of Biomedical Sciences (ICB-I), University of São Paulo (USP), Avenida Prof. Lineu Prestes 1524, Butantã, 05508-000 São Paulo, SP, Brazil; ^4^University of Toronto, Tanz Centre for Research in Neurodegenerative Diseases, Department of Medical Biophysics, Krembil Discovery Tower, 60 Leonard Avenue, Toronto, ON, Canada M5T 2S8

## Abstract

Type 2 diabetes (T2DM), Alzheimer's disease (AD), and insulin resistance are age-related conditions and increased prevalence is of public concern. Recent research has provided evidence that insulin resistance and impaired insulin signalling may be a contributory factor to the progression of diabetes, dementia, and other neurological disorders. Alzheimer's disease (AD) is the most common subtype of dementia. Reduced release (for T2DM) and decreased action of insulin are central to the development and progression of both T2DM and AD. A literature search was conducted to identify molecular commonalities between obesity, diabetes, and AD. Insulin resistance affects many tissues and organs, either through impaired insulin signalling or through aberrant changes in both glucose and lipid (cholesterol and triacylglycerol) metabolism and concentrations in the blood. Although epidemiological and biological evidence has highlighted an increased incidence of cognitive decline and AD in patients with T2DM, the common molecular basis of cell and tissue dysfunction is rapidly gaining recognition. As a cause or consequence, the chronic inflammatory response and oxidative stress associated with T2DM, amyloid-*β* (A*β*) protein accumulation, and mitochondrial dysfunction link T2DM and AD.

## 1. Introduction

In the last few decades changes in lifestyle, especially related to overnutrition, physical inactivity, and ageing have increased the global incidence of Type 2 diabetes (T2 DM). According to the International Diabetes Federation (IDF), currently 387 million people have diabetes mellitus worldwide, and this number is expected to reach 592 million by 2035. T2DM is by far the most common form of diabetes, representing about 90–95% of DM cases. In older age people (>65 years), the prevalence of T2DM is 12–25% and is characterized by cell and tissue insulin resistance, metabolic dysregulation, and chronic inflammation. These clinical abnormalities have also been described in dementia cases [1, 2]. Hoyer first suggested the concept of disturbances in glucose metabolism and insulin resistance as underlying causes of neurodegeneration and dementia [3, 4].

More recently, epidemiological studies have provided further evidence for this link where T2DM was shown to be associated with accelerated cognitive decline and increased risk of dementia (by 1.5- to 2-fold). Indeed 10% of world-wide cases of dementia maybe attributable to the metabolic disturbances associated with T2DM [5]. Moreover, cross-sectional and longitudinal studies have indicated that global brain atrophy is strongly associated with T2DM and the rate of atrophy is greater than that seen in normal ageing [6, 7]. Very recently in the Framingham Heart study it was reported that diabetes, and in particular the resulting hyperglycaemia, was associated with reduced cognitive performance and reduced brain grey matter volume in young and middle-aged adults [8].

Strengthening the relationship between diabetes and reduced cognition, it can be postulated that metabolic changes associated with diabetes can potentially drive early neurodegenerative processes in dementia. The molecular underpinnings of this relationship are both complex and poorly understood. The purpose of this review is to examine the common and disparate metabolic, oxidative, and inflammatory processes with the aim of collating in one place current understanding linking T2DM with the most common cause of dementia, Alzheimer's disease (AD).

## 2. The Involvement of Chronic Inflammation in Insulin Resistance, Obesity and Diabetes

**Figure 1:**
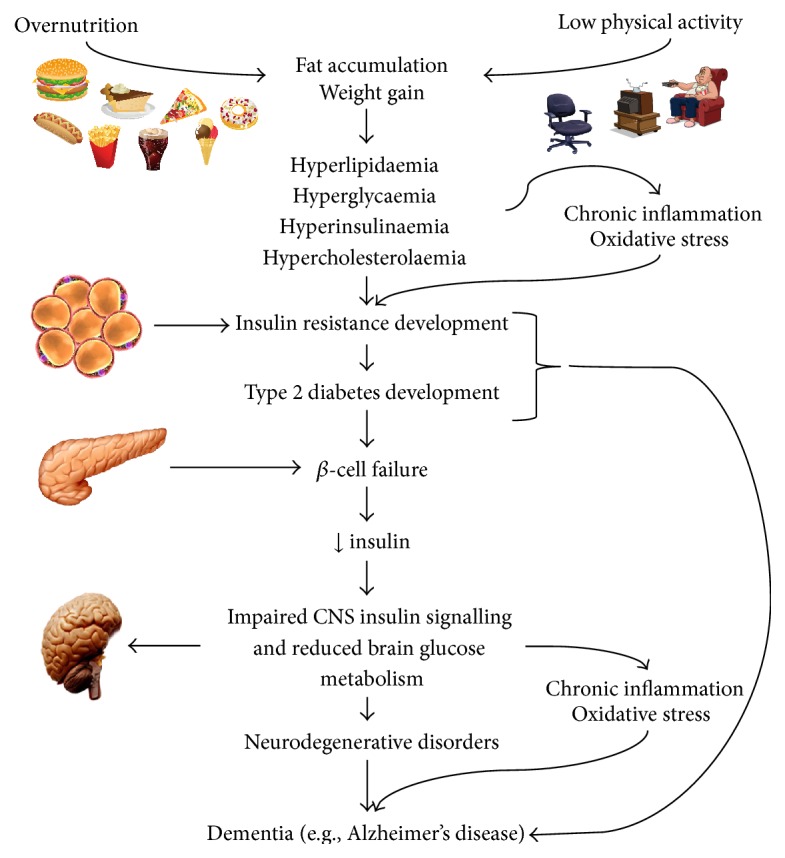
Neurodegeneration, insulin resistance, obesity, and T2DM. Metabolic overload, chronic inflammation, and oxidative stress promote cellular dysregulation in both T2DM and AD. Brain IR may occur in the absence of diabetes suggesting that AD may develop in the earlier stages of insulin resistance. Chronic inflammation and oxidative stress are considered two key factors linking diabetes and AD [2].

Obesity and related metabolic diseases are associated with chronic low-grade inflammation (Figure 1). In 1993, the relationship between obesity and T2DM was described* in vivo*, when investigators demonstrated that adipose-derived tumour necrosis factor-*α* (TNF-*α*) levels in mice were increased during the development of obesity [9]. When TNF-*α* was neutralised, insulin sensitivity was improved [9], linking adipose tissue inflammation with insulin resistance. In normal circumstances, interaction of insulin with insulin receptor (IR) will promote conversion of fatty acids into triacylglycerols, using glucose-derived glycerol 3-phosphate as a substrate for esterification, in adipocytes. Consequently, insulin induces a coordinated uptake of fatty acids and glucose into adipose tissue, followed by esterification into triacylglycerol. Obviously, any dysregulation of these pathways will lead to excess levels of circulating glucose and fatty acids, which is observed in T2DM.

Interestingly, adipose tissue also releases adipocytokines (adipokines) into the circulation, such as leptin and adiponectin. In 1994, leptin was shown to modulate food consumption and energy expenditure via neuroendocrine signalling in the hypothalamus [10]. Comparably, adiponectin was demonstrated to promote improved insulin sensitivity, and mice with adiponectin-deficiency were severely insulin resistant [11]. Thus, adipokines are believed to modulate insulin sensitivity in the principal organs targeted by insulin, such as liver, adipose, and skeletal muscle.

As a component of the development of obesity and related metabolic dysfunction, immune cells such as macrophages accumulate in the adipose tissue, secreting proinflammatory cytokines that impact glucose and lipid metabolism [11]. The infiltration of excessive numbers of macrophages into adipose tissue and subsequent activation is critical to TNF-*α* and interleukin- (IL-) 6 production and release, which can reduce lipoprotein lipase enzyme activity, thus increasing blood lipid levels. In parallel, hormone-sensitive lipase activity can be enhanced in adipose tissue by TNF-*α*, which further increases the release of nonesterified fatty acids (NEFAs) into the blood, while concomitantly reducing insulin-stimulated glucose uptake via impaired insulin signalling. Taken together, these effects would significantly increase plasma lipid levels and, in combination with reduced lipid disposal by adipose tissue, would lead to damaging levels of blood LDL in T2DM. In addition, other proinflammatory cytokines can negatively affect metabolic pathways responsible for correctly storing/oxidising glucose and lipids in tissues that are physiological targets for insulin. These inflammatory exchanges may lead to hyperglycaemia and dyslipidaemia, which are important parameters indicative of insulin resistance, obesity, and T2DM [6, 11] (Figure 1).

## 3. Mechanisms Driving Peripheral Insulin Resistance

Insulin resistance is a phenomenon that plays a significant role in the progression and development of metabolic diseases associated with neurodegeneration and obesity (Figure 1). Insulin resistance refers to impaired or failed cell response to insulin receptor-activated signalling in insulin-sensitive tissues such as the liver, skeletal muscle, adipose [12], and brain [2, 13]. This results in a reduction of glucose uptake by these tissues, with a concomitant increase in hepatic glucose output, both leading to elevated plasma glucose concentrations. The subsequent change in glucose homeostasis places an increased burden on pancreatic *β*-cells to produce and secrete more insulin in order to restore normal blood carbohydrate levels. While this compensatory mechanism may alleviate glucose levels in early or prediabetes, persistent insulin resistance and continued exposure of *β*-cells to excess blood glucose and lipids promote *β*-cell dysfunction, failure, and ultimately death, culminating in overt diabetes (Figure 1).

Once released into the circulation by *β*-cells in response to elevated blood glucose levels, insulin elicits its anabolic effects via association with the transmembrane insulin receptor (IR) in target tissues. The interaction with insulin induces autophosphorylation of the receptor and the recruitment and phosphorylation of insulin receptor substrate (IRS) proteins and activation of associated downstream signalling cascades, for example, phsosphatidylinositol-3-kinase (PI3K) and protein kinase B (Akt) [14, 15]. Akt is an important regulator of GLUT-4 vesicle translocation to the plasma membrane, which is critical for the intracellular uptake of free glucose in insulin-sensitive tissues [16, 17]. Further details and a full description of nutrient regulated insulin action have recently been published [12].

Insulin resistance can occur due to interference in the common insulin signalling cascade due to either genetic mutations or structural modifications to any of the signalling nodes in the insulin signalling pathway. In particular, mutations and serine associated hyperphosphorylation of IRS proteins have been linked with development of insulin resistance and it was speculated that this is because of decreased interaction with PI3K [18]. Homozygous interruption of IRS1 expression in mice led to mild insulin resistance [19], while complete depletion of IRS2 expression in rodents resulted in severe insulin resistance [20].

Structural modification by hyperphosphorylation of serine at residues Ser^302^, Ser^307^, Ser^612^, and Ser^632^ in IRS1 was suggested to be an important mechanistic element responsible for increased insulin resistance in rodent models [18]. Indeed, excessive expression of proinflammatory cytokines and signalling proteins such as TNF-*α* and JNK1, which may be derived from adipose expansion, can induce serine hyperphosphorylation of IRS1 [21, 22], particularly at residue Ser^636^. However, it is not entirely clear which individual residues or combination of residues must be hyperphosphorylated to promote the insulin resistant phenotype.

Another possible molecular mechanism that may lead to impaired insulin signal transduction is the dysfunctional regulation of PI3K activity. It was previously suggested that elevated expression of individual regulatory domains of PI3K (e.g., p85) in skeletal muscle, promoted pregnancy-induced insulin resistance by preventing the binding of the signalling heterodimer with IRS [23]. Conversely, genetic deletion of PI3K p85 regulatory domains in the liver of mice boosted hepatic and peripheral insulin sensitivity [24].

On the other hand, the insulin resistant phenotype may be a consequence of a more direct mechanism that promotes decreased IR expression or desensitisation to the insulin ligand. It is speculated that chronic hyperglycaemia and prolonged hyperinsulinaemia, along with increased reactive oxygen and nitrogen species (ROS and RNS, resp.) levels may affect IR gene expression via dysfunction of key transcription factors such as high mobility group AT-hook 1 (HMGA-1) [25] or may induce IR-desensitisation which under normal conditions is a process under negative-feedback control [26]. Chronically, hyperinsulinaemia is a key pathological characteristic of insulin resistance but it is not clear whether this is a cause or a consequence [26, 27].

Interestingly, excessively high carbohydrate levels can also promote decreased insulin binding and reduced IR mRNA expression in skeletal muscle [28]. High glucose and high insulin in combination may reduce insulin binding to the IR in adipocytes [29], resulting in a negative impact on Akt activity. Increased production of ROS/RNS or decreased antioxidant capacity as a result of increased carbohydrate metabolism in insulin target tissues may alter the phosphorylation status of these signalling nodes causing deactivation. It has been shown that oxygen peroxide (H_2_O_2_) exposure can induce a significant loss in proximal and distal insulin signalling along with decreased glucose transport in adipocyte and muscle cell lines* in vitro* [17].

**Figure 2:**
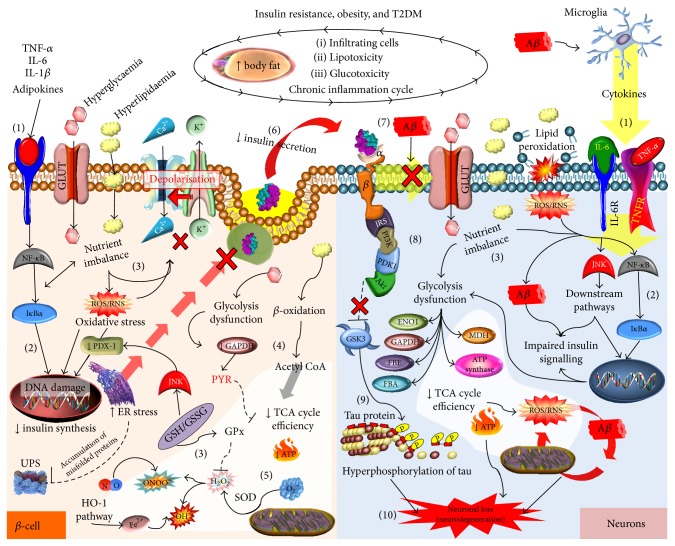
Molecular mechanisms linking insulin resistance with neurodegeneration. Obesity is characterized by chronic low-grade inflammation, which impacts all tissues and organs. Inflammatory cytokines bind to their receptors (1) activating the nuclear factor-kappaB (NF-*κ*B/I*κ*B*α*) pathway, which stimulates a proinflammatory condition (2). Nutrient imbalance may also activate inflammatory pathways and DNA damage, adversely impacting redox regulation (via glutathione peroxidase (GPx); glutathione (GSH); and oxidised glutathione (GSSG) levels) and so promoting oxidative stress (3). *β*-cell metabolism and ATP production are affected by nutrient imbalance via glycolytic dysfunction and reduced activation of glyceraldehyde 3-phosphate dehydrogenase (GAPDH) reducing pyruvate (PYR) generation but promoting *β*-oxidation (4). As a result of the metabolic dysfunction, superoxide and subsequently hydrogen peroxide generation (which can combine with nitric oxide, NO, to create peroxynitrite, an example of RNS) may occur due to compromised mitochondrial electron transport chain, ETC, and action, and so reducing ATP synthesis (5). All these processes impact endoplasmic reticulum (ER) stress, leading to a reduction in the ability to secrete insulin (6). High circulating levels of lipids and glucose and chronic inflammation increase amyloid beta (A*β*) aggregation, which together with low insulin reduce the transport and utilisation of glucose in the brain (7) via impairment in insulin signalling (8), including the negative regulator of glycogen synthase kinase 3 (GSK3). Activated GSK3 is associated with tau hyperphosphorylation (9). The vicious cycle mediated by ROS/RNS and A*β* may eventually result in enzyme inhibition (e.g., alpha-enolase (ENO1), malate dehydrogenase (MDH), ATP synthase, and GAPDH), lowering ATP generation, which together with tau promotes neuronal loss (10). Protein kinase B (AKT); fructose bisphosphate enolase (FBE); fructose bisphosphate aldolase (FBA); calcium (Ca^2+^); iron (Fe^2+^); glucose transporters (GLUT); hydrogen peroxide (H_2_O_2_); interleukin- (IL-) 1 and interleukin- (IL-) 6; insulin receptor substrate (IRS); Janus kinase (JNK); potassium (K^+^); nitric oxide (NO); anion superoxide (O_2_^−^); hydroxyl radical (OH^−^); peroxynitrite (ONOO^−^); pyruvate dehydrogenase kinase, isozyme 1 (PDK1); pancreatic and duodenal homeobox 1 (PDX-1); Phosphatidylinositol-4,5-bisphosphate 3-kinase (PI3K); superoxide dismutase (SOD); tumour necrosis factor alpha (TNF-*α*); ubiquitin-proteasome system (UPS).

More specifically, H_2_O_2_ promoted Ser^307^ phosphorylation of IRS1 and this led to the enhancement of IRS proteolysis [30, 31]. Indeed, proinflammatory cytokines such as TNF-*α* can also promote phosphorylation of similar IRS residues and thereby decrease the interaction of the protein with the IR. Proinflammatory cytokines are elevated in obesity and T2DM and when coupled with excessive oxidative stress maintain a proinflammatory environment, which leads to further activation of proinflammatory pathways (NF*κ*B and JNK) and enhances the recruitment of immune cells to insulin target tissue. Consequently, inflammation also plays a significant role in T2DM progression and insulin resistance (Figure 2).

## 4. Insulin Resistance Can Result in the Development of Neuronal Dysfunction in Alzheimer's Disease (AD)

As in peripheral tissues, insulin through its action on the hypothalamus has important roles in the brain to regulate appetite, glucose, and lipid homeostasis [32, 33]. Insulin and its receptor are also abundant in other areas of the brain including the hippocampus [34] where insulin signalling is becoming increasingly recognised to have function in modulating memory and learning and being required for synaptic plasticity [35, 36] and neuronal stem cell activation and has neuroprotective properties [37, 38]. Although not the main function in the brain, insulin can promote glucose uptake through its regulation of GLUT-4 transporter (GLUT-4) [39]. But GLUT-4 is only expressed in certain neurons (i.e., hippocampal neurons) [40] and is not considered as the primary transporter of glucose in the brain. This role is primarily carried out by the major GLUTs expressed in the brain, GLUT-1 (in astrocytes) and GLUT-3 (neurons), independently of insulin [41]. The disruption of insulin signalling and glucose transport in neurons can contribute to the progression of neurodegenerative diseases such as AD (Figure 2).

Peripheral hyperinsulinaemia, but reduced insulin sensitivity in fasting and during glucose tolerance tests, has been reported in AD patients [1]; however, the impact on insulin levels and sensitivity in CNS remain to be fully determined. An initial study by Fujisawa and colleagues showed that compared to controls both peripheral and CSF insulin levels were higher in AD subjects following a glucose tolerance test and fasting [42]. Subsequent studies have either shown a reduction [43] or no change [44] in fasting CSF insulin levels in AD subjects compared to controls. As discussed by Reger and colleagues, differences in potential confounding factors such as BMI and control for AD genetic risk factors (i.e., presence of APOE*ε*4) or AD severity may have accounted for differences between these studies. More recently, findings from Suzanne Crafts group have shown that CSF insulin levels are reduced in early stages of AD or in the mild cognitively impairment [45] and diets rich in fats and sugar lower CSF insulin levels in healthy adults and this was associated with reductions in cognitive functioning [46]. Whether changes in peripheral insulin levels reflect changes in brain insulin levels is unclear. However, it is possible that increases in peripheral insulin levels acutely elevates CSF and brain insulin levels, but prolonged hyperinsulinaemia reduces insulin transport to the brain, by downregulating insulin receptors at the BBB [47].

Cerebral glucose metabolism is reduced in AD brain and FDG-PET imaging studies have shown that this is an early feature of disease progression [48]. In imaging studies of AD pathology (i.e., PET amyloid imaging), associations with hyperglycaemia, insulin resistance, and cerebral glucose hypometabolism are less clear with conflicting findings among studies [49–52]. Factors such as age of disease onset, stage of disease (preclinical, early, and late), co-morbidities, different populations, cohort sizes, and type (longitudinal versus cross sectional) require consideration in future studies. Despite this,* in vitro* and* in vivo* animal studies have provided insight into the associations between T2DM and AD pathology.

Key pathological hallmarks of the AD brain include brain atrophy (due to neuronal loss), extracellular deposition of amyloid plaques, accumulation of intracellular neurofibrillary tangles (NFTs), inflammation, and oxidative stress. Amyloid plaques result from the accumulation of the amyloid-*β*-protein (A*β*). Together with A*β*, the accumulation of the major component of NFTs, hyperphosphorylated tau protein, is thought to drive neurodegeneration [53, 54]. The accumulation and deposition of A*β* in the brain is thought to occur early in the disease process [55] and initiate downstream events, including tau phosphorylation, inflammation, and oxidative stress that leads to neurodegeneration. The accumulations of nonfibrillar, soluble small aggregates (“oligomers”) of A*β* (rather than larger aggregates/plaques) are major contributors to neurotoxicity where they inhibit synapse formation, impair memory and learning in animal models, and correlate well with the severity of neurodegeneration [56, 57]. Although underlying mechanisms remain to be fully elucidated, A*β* accumulation can alter number of cellular processes resulting in neuronal dysfunction [57], including brain insulin signalling.

As demonstrated in T2DM for muscle or adipose tissue, the ability of insulin to activate specific signalling pathways is weaker than normal in the AD brain. Brain levels of insulin and binding to the insulin receptor are reduced with age [58] and are markedly reduced in the AD brain compared to the controls [59, 60]. The accumulation of A*β* oligomers can inhibit the autophosphorylation of the insulin receptor (IR) [61]. Oligomers can also markedly reduce IR levels and activity within dendrites of hippocampal neurons [62], leading to dendritic and synaptic loss [63]. Both* in vitro* [63] and* in vivo *[13] studies have shown that IR loss and subsequent loss of synapses can be prevented through administering insulin. The loss of IR expression or impaired activity has many downstream cell signalling consequences.

Studies have shown elevated serine phosphorylation of IRS1 [2, 64, 65] resulting in the inability to transmit signals to secondary messengers, such as PI3K as described above [66]. This has downstream effects on other brain pathological markers including tau phosphorylation and neuroinflammation. PI3K/Akt signalling can mediate a number of downstream pathways including Wnt/*β*-catenin pathway [67], mTOR signalling [68], and regulating GSK3*β* activity. GSK3*β* is a kinase involved in the phosphorylation of tau and the deficiencies in PIK3 signalling are thought to lead to a reduction in Akt signalling resulting in reduced ability to regulate GSK3*β* activity, thereby promoting tau hyperphosphorylation and the formation of NFTs [69, 70] (Figure 2). However, very recent findings argue against this notion [71] but show that instead of deficiencies in signalling, an upregulation of PI3K/Akt/mTOR pathway occurs in brain tissue from AD and MCI subjects. In addition, compared to control brain, the authors found that GSK3*β* expression was reduced in AD brain and reduction in Ser9 and increase in Tyr216 phosphorylated GSK3*β* were also observed in AD brain. Phosphorylation of Ser9 or Tyr216, respectively, can attenuate or stimulate GSK3 *β* activity [72–74]. Therefore, the findings by Tramutola and colleagues are in contrast to the notion that overactivation of GSK3*β* leads to tau hyperphosphorylation and questions strategies to inhibit/attenuate GSK3*β* as therapeutics for AD as to date inhibitors have shown little benefit in clinical trials [75].

A*β*-mediated phosphorylation of Akt can also occur in the absence or presence of insulin [62], suggesting the involvement of IR-independent pathways, possibly through inflammatory cytokines. Proinflammatory cytokines TNF-*α*, IL-1*β*, and IL-6 are increased in both T2DM and AD [76] and this can have neurotoxic effects in the CNS [77]. This increase could exacerbate the effects observed from the activation of microglia by A*β* oligomers which promotes the secretion of further proinflammatory cytokines. The accumulation of these cytokines can induce neuronal death by increasing apoptosis, reducing synaptic activity, and inhibiting neurogenesis [78].

One potential mechanism by which inflammation can potentiate its neurotoxic effects is through blocking of intracellular actions of insulin, as proinflammatory cytokines can activate kinases to phosphorylate IRS1 at specific serine residues associated with downstream inhibition of insulin signalling events [79]. Other mechanisms could involve the receptor for advanced glycation end products (RAGE), which in the CNS is expressed in neuronal cells, microglia, astrocytes, and brain endothelial cells. RAGE levels are increased in AD and T2DM [80] and are a potential mechanism for vascular dysfunction [81] in these diseases and interactions between disturbed glucose metabolism, oxidative stress, and accumulation of AGEs are important in the vicious cycle that contributes to AD progression and T2DM [82]. In addition to its role in transport of A*β* across the BBB from the periphery into the brain [83] which can promote neurodegenerative pathways, its expression in many cell types within the CNS and binding to A*β* can induce cerebrovascular dysfunction and promote the release of cytokines (TNF-*α* and IL-6) from microglia, potentially through inducing the expression in neurons of macrophage-colony stimulating factor (M-CSF) [83] and subsequent neuronal damage [81], further perpetuating the vicious cycle. RAGE-ligand interactions can lead to BACE1 expression, promoting the amyloidogenic processing of APP and more A*β* generation [84]. The BACE1 promoter also contains an NF-*κ*B binding site [85] which is activated during RAGE-ligand interaction [86], leading to enhanced expression of RAGE resulting in further oxidative stress and inflammation which in turn sustains the formation of advanced glycation products (AGEs), A*β*, and impaired insulin signalling [87].

There is also evidence that suggests that the RNA-dependent protein kinase (PKR) is a critical mediator of the inflammatory response in insulin resistance [88–90] and in AD [91–94]. The proapoptotic kinase, PKR, controls the initial step in protein translation and modulates cell death and survival pathways. It is an important regulator of the production of proinflammatory factors through the activation of NF-*κ*B [95] and in the control of the inflammasome [96]. Inducing systemic inflammation (via administering LPS) can promote brain neuroinflammation increase A*β* production and PKR phosphorylation, which is downregulated in PKR knockdown mice [93], providing evidence for a role of PKR signalling in AD pathogenesis. The PKR signalling pathway and eIF2a phosphorylation may also be a potential molecular link between T2D and neurodegeneration that occurs in AD (recently reviewed in [97]). Lourenco and colleagues (2013) [94] showed that phosphorylated PKR and its target eukaryotic translation initiation factor 2a (eIF2*α*) were increased in brains of AD mice. Exposure of neurons to A*β* oligomers or administering A*β* oligomers to monkeys via i.c.v. also increased phosphorylation of PKR and eIF2*α*. In mice lacking PKR or TNF receptor or mice treated with a TNF-*α* neutralising antibody, A*β* oligomers failed to induce phosphorylation of PKR and eIF2*α*. Further, activation of the TNF-*α*/PKR/eIF2*α* pathway was linked to synapse loss and memory impairment in mice. Insulin, or glucagon-like peptide 1 (GLP-1) receptor agonists inhibited A*β* induced phosphorylation eIF2*α*, indicating that stimulating insulin signalling may prevent inflammation mediated synaptic loss and memory impairment through inhibiting/downregulating the PKR signalling pathway.

Failure of the endoplasmic reticulum (ER) adaptive capacity and subsequent activation of the unfolded protein response (i.e., ER stress) intersects inflammatory and other stress pathways (reviewed in [89]) and plays a key role in the pathogenesis of both T2D and AD. In metabolic disorders, such as obesity, inflammatory mediators and lipids can activate signalling cascades that trigger inflammatory mediators such as JNK and IKK. This in turn can lead to serine phosphorylation of IRS1/2 and subsequent inhibition of insulin signalling [89]. The activation of inflammatory signalling pathways can trigger ER stress which can lead to further inhibition of insulin action in addition to generation of reactive oxygen species (ROS) through mitochondrial dysfunction [89].

In the AD brain, markers of ER stress including BiP/GRP78, pERK, and eIF2*α* are elevated [98, 99], and expression of PKR-p is increased in AD brain [100]. A*β* oligomers can upregulate ER stress responses [94, 101] potentially through activating PKR signalling and phosphorylation of eIF2a via TNF-*α* pathway [94]. Ab can also lead to synthesis and transport of the ATF4 transcription factor within neuron axons, a potential mechanism by which neurodegenerative signals are transmitted between neurons and promote spreading of AD pathology and neurodegeneration [102]. Further, eIF2*α*-P can promote BACE1 expression and subsequent A*β* production [103], suggesting that a feed-forward cycle may perpetuate further stress and neuronal dysfunction. Targeting these stress pathways may offer therapeutic targets in AD. This is highlighted by recent work in which the conditional knockout of eIF2a kinases (PERK and GCN2) prevented A*β* induced impairment in long term potentiation (LTP) (synaptic activity) and memory impairment in AD transgenic mice [104]. This is further supported by findings that insulin and GLP-1 receptor agonists reduces eIF2*α* phosphorylation and prevents A*β*-mediated neuronal dysfunction [94].

### 4.1. Insulin Resistance Is Associated with Elevated A*β*

The features of insulin resistance in T2DM including hyperglycaemia, dyslipidaemia, and hyperinsulinaemia are all known to promote A*β* accumulation [105–109] (Figure 2). It is difficult to tease apart which of these metabolic disturbances contribute as single or in combination with the development of neuronal dysfunction, which disrupts the production or clearance of A*β*. Importantly, an excess of A*β* accumulation in the brain establishes a vicious cycle of impaired brain insulin signaling, inflammation, and oxidative stress processes that promote neurodegeneration in the AD brain. There is also some evidence that A*β* accumulates in peripheral tissues such as the pancreas [107, 110] and that A*β* can induce insulin resistance in the liver [111] suggesting that not only can insulin resistance promote A*β* accumulation but also the reverse could occur. Further confirmation for this interesting scenario is required and in particular determining if this is a cause or effect of insulin resistance/A*β* accumulation.

Studies of high fat feeding in transgenic mouse models of AD or diabetic rodent models have shown that insulin resistance can lead to an increase in the expression of key enzymes that generate A*β* (BACE1 and *γ*-secretase) [107, 108]. Upregulation of the autophagy pathway has also been shown to contribute to A*β* accumulation, where autophagosomes are sites of A*β* generation [112] and accumulate following insulin resistance [108]. This could result from defects in insulin signalling resulting in deficiencies in autophagic flux (and removal of autophagosomes), resulting from the inhibition of the signalling target molecule mTOR [108].

In sporadic AD cases, impaired clearance or removal of A*β* from the brain is a major contributor to promoting amyloid accumulation. The removal of A*β* from the brain can occur via a number of mechanisms, including promoting the efflux of A*β* from the brain and enhancing the degradation of A*β* (for review see Bates et al. [113]). Degradation by the insulin degrading enzyme (IDE) has been suggested to be the primary regulator of A*β* [114, 115], where overexpression has shown to dramatically reduce A*β* accumulation [116] and depletion/reduction in levels has shown to promote A*β* accumulation [117, 118]. Through competitive inhibition of IDE increased insulin levels can inhibit IDE leading to A*β* accumulation in the periphery and CNS [119, 120]. In addition, depletion of IDE levels or reduced activity leads to hyperinsulinaemia and impaired glucose tolerance associated with a chronic elevation of A*β* [117]. Overall these findings in various* in vivo* models suggest that reduced expression or activity of IDE is a major contributor to the A*β* accumulation and development of AD pathology.

In line with this notion are studies that show reduced IDE levels in AD patients compared to controls [114, 121, 122]. A more recent study argues that in addition to enhancing A*β* production, T2DM impairs the clearance of A*β* but not through altering IDE expression [107, 123]. AD transgneic mice fed a high fat diet showed gluose intolerance associated with inuslin resistance and impaired insulin production associated with accumulation of A*β* in the brain and the periphery. This accumulation of A*β* and associated memory impairments was reversed with the acute administration of insulin [107]. Brain levels of IDE or transporter proteins (LDL receptor-related protein 1 and RAGE) invovled in the efflux/influx across the blood-brain-barrier were not altered. Instead, insulin administration led to an increase of blood A*β* associated with a reduction in brain A*β*, indicating clearance of A*β* into the blood. The authors concluded that a combination of complementary mechanisms of CNS A*β* production and clearance towards the blood underlies the benefits of insulin at reversing AD pathology in mice in this study. However, it is unclear how A*β* is transported across the blood-brain-barrier and into the blood, and the authors did not explore the role of other A*β* transporters including apolipoprotein J [123].

To attempt to tease out the effects of elevated blood glucose levels independent of insulin resistance, a very recent study combined glucose clamps and* in vivo* microdialysis to assess changes in AD transgenic mice during a hyperglycemic challenge [109]. The authors found that increased blood glucose levels as a result of the clamp were associated with and increased A*β* levels in the interstitial fluid (ISF) in young mice and persisted after euglycaemia was restored. Whilst total A*β* load in the brain did not change, hippocampal metabolism and neuronal activity were reduced. This effect was exacerbated in older mice with established plaque pathology, indicating that age and pathology can influence the brains response to the metabolic insults. The study also suggests that repeated exposures to acute hyperglycaemia can promote A*β* accumulation altering hippocampal and neuronal functioning early in the disease process. This is consistent with a recent study that showed that increased fasting blood glucose was associated with reductions in brain gray matter and hippocampal volume and was also associated with impaired attention and memory in young and middle-aged adults [8]. A graded association was observed between fasting blood glucose levels in normal, prediabetic, and diabetic ranges and measures of brain atrophy. This has clinical relevance as these studies suggest that even at an early stage of diabetes (or prediabetes), increases in blood glucose levels have detrimental effects on memory, hippocampal integrity, and A*β* accumulation. In addition, there are conflicting results amongst other studies that assess improvements in memory following glycaemic control in the elderly. For example, in the ACCORD memory study of elderly patients with T2DM, intensive glycaemic control showed a small difference in brain volume but no evidence of cognitive improvement and furthermore the targeted treatment was associated with increased mortality [124]. Taken together, these studies demonstrate that age, duration, and severity of pathology impact the effects of diabetes on the brain and that treatment in late-life may be not as effective as preventative strategies that can be implemented at young age.

## 5. Redox Regulation Pathways: Common Targets in T2DM and AD

Dysregulation of some metabolic, molecular, and cellular processes is common in T2DM and AD, particularly in *β*-cells and neurons, respectively. Cell and tissue oxidative stress are a key player in both diseases. Pathophysiologically, reactive oxygen species (ROS) and reactive nitrogen species (RNS), such as superoxide anion (O_2_^∙−^), hydrogen peroxide (H_2_O_2_), hydroxyl radical (OH^∙^), nitric oxide (NO), and the related peroxynitrite (ONOO^−^), contribute to key metabolic and physiologic processes. This includes mitochondrial function [125], which if impaired will reduce ATP generation capacity which will impact *β*-cell glucose stimulated insulin secretion (GSIS), the NADPH complex, and Ca^2+^ signalling associated with neurotransmission [126, 127].

With respect to insulin resistance and the progression to T2DM, continuous overnutrition leads to chronic ROS and RNS production, which promotes oxidative stress in key cells, tissues, and organs. Eventually, oxygen and nitric oxide based free radicals damage cell membranes, DNA, and protein structures, as well as modulating the activity of transcriptional factors through redox chemistry, including NF-*κ*B, leading to chronic inflammation and cell apoptosis [125]. Although every single cell can be potentially damaged by oxidative stress, the reduced capacity of peroxidase based antioxidant defence mechanisms can particularly expose both *β*-cells and neurons to damage resulting in progression of T2DM and AD (Figure 2). The sources of ROS from mitochondrial dysfunction, inflammation, advanced glycation end products (AGEs), and increased cytosolic Ca^+2^ levels can promote redox dysregulation and perpetuate oxidative stress in AD and T2D (recently reviewed in [128].

The reduced capacity to scavenge free radicals is a major avenue for increased ROS production. Glutathione (GSH) is one such important free radical scavenger that is generated from the reduction of glutathione disulphide (GSSG) by the enzyme glutathione reductase. In addition to many other reducing enzymes, NADPH is an essential cofactor for the activity of glutathione reductase. Other metabolic pathways that are favoured under conditions of overnutrition or hyperglycemia can consume NAPDH, thereby reducing the cells capacity to generate GSH. One example is the polyol pathway flux in which glucose is reduced to sorbitol by aldose reductase; sorbitol is then transformed to fructose by sorbitol dehydrogenase. Although aldose reductase has a low affinity for glucose, activity is increased under hyperglycemic conditions and thus more NADPH is consumed [128]. In addition, reduced levels of GSH can result from T2D, due to impaired protein turnover or dietary deficiency in essential amino acids required to synthesize GSH [129]. Similarly, GSSG levels are reduced in AD patients and correlate with reduced cognitive functioning [130, 131] and have been investigated as potential for a biomarker [132]. Inhibiting the polyol pathway has been shown to normalise sorbitol in the brain in the presence of hyperglycaemia [133], suggesting that impaired cognitive functioning associated with hyperglycaemia may be attenuated through preventing the breakdown of sorbitol (and thus minimising the consumption of NADPH).

The conversion of glucose to ribose-5-phosphate by glucose-6-phosphate dehydrogenase (G6PD) is the first and rate-limiting step of the pentose phosphate pathway and is a major pathway that generates NADPH. A decrease in G6PD activity can thereby lead to a reduction in NADPH, promoting oxidative stress [134–136]. This reduction in activity can in part be induced by the increased phosphorylation of G6PD by protein kinase A (PKA) activation under high glucose conditions [137]. Interestingly, an increased PKA activity has been associated with tau phosphorylation [138, 139] and impaired synaptic activity [36, 140, 141].

Other sources of NADPH include those generated from isocitrate dehydrogenase (IDH) activity within the citric acid cycle that converts NADP+ to NADPH and malic enzyme which catalyses the formation of pyruvate, CO_2_, and NADPH from malate and NADP+. When exposed to high glucose, glucose 6-phosphate, and fructose, IDH undergoes fragmentation and carbonylation leading to reduced activity [142]. This reduction correlated with ROS generation, DNA fragmentation, lipid peroxidation, and decreases in ATP levels [143]. In the liver, malic enzyme supplies NADPH for fatty acid biosynthesis, but in the brain mainly located in oligodendrocytes malic enzyme generates NADPH for myelin lipid synthesis for myelination of neuronal axons (Rosales-Corral et al., 2015). Malic enzyme may also serve to regenerate GSH in the brain as the enzyme is abundant in mitochondria of neurons [144–146]. In adipose tissue the levels of ME are diminished in T2D, linked to reduced lipogenesis [147]; however, more recently, although there were reductions in other NAPDH synthesising enzymes, levels of malic enzyme in islet pancreatic cells were not significantly different in diabetics compared with controls [148]. Overall, the failure to replenish endogenous antioxidants such as GSH can occur due to diminished NADPH levels due to a high demand in its utilisation in a number of pathways that are promoted with T2D. This in turn diminishes the capacity to scavenge ROS, favouring oxidative stress and progression in T2D/AD.

The capacity to reduce oxidised proteins also plays a role in promoting oxidative stress processes and this is regulated by a number of proteins that act as redox sensors. A major class of these redox proteins is thioredoxin (Trx) which reduce oxidised proteins by cysteine thiol disulfide exchange [149]. In the process, Trx is oxidised which is further reduced by NADPH. Trx can be inactivated by alkylating agents or, as in T2D, the oxidative stress mediator thioredoxin-interacting protein (TxNIP), which is upregulated by glucose [150]. TxNIP can mediate glucotoxicity in islet cells [151] and trigger activity of the NLRP3 inflammasome [152] and can promote ER stress and apoptosis. TxNIP expression is also induced in neurons after oxidative stress, ER stress, or ischemic injury ultimately resulting in neuronal death [153, 154]. TxNIP is also overexpressed in brains of and AD mouse model and can be induced by A*β*,* in vitro* [155], suggesting a role for TxNIP in AD pathogenesis.

The GSH/GSSG and thioredoxin pathways are examples by which proinflammatory processes are aided by redox sensors which are regulated by changes in redox potential and are modulated by physiological or pathological situations. Under conditions of T2D/AD where oxidative stress features and persists the redox reduction of redox sensors diminishes and shifts cells toward proinflammatory pathways promoting apoptosis and more oxidative stress establishing a vicious cycle that ultimately leads to cell dysfunction/death.

### 5.1. *β*-Cell Oxidative Stress

Pancreatic *β*-cell responsiveness to glucose, the major insulin secretagogue, is dependent on the acute regulation of intracellular and, in certain cases, extracellular ROS and RNS [126, 127]. Increased glycolytic flux boosts oxidative phosphorylation and ATP production but may also lead to O_2_^∙−^ formation and release from the electron transport chain [156]. In addition, conversion of surfeit glucose to pentose via the pentose phosphate pathway is an initial adaptive response to deviate glucose carbon away from excessive glycolysis and oxidative phosphorylation, but shuttling glucose in this direction may also promote NADPH oxidase (NOX) activity leading to increased O_2_^∙−^ synthesis. Importantly, high glucose levels raise ROS via other mechanisms, such as glucose autoxidation and generation of AGEs.

Removal of O_2_^∙−^ requires the action of superoxide dismutase (SOD), generating the more stable but perhaps less directly damaging H_2_O_2_. However, H_2_O_2_ can subsequently generate the highly reactive OH^−^ by the iron-catalysed Fenton reaction [126, 156, 157] and with nitric oxide (NO) forms ONOO^−^ [126]. ROS and RNS cause oxidative damage to DNA, lipids, and proteins through nitration, carbonylation, peroxidation, and nitrosylation reactions. These molecular modifications may alter enzyme activity, ion channel transport, or receptor signal transduction and consequently dysregulate gene expression, which may impact *β*-cell functionality [126]. Moreover, ROS-mediated activation of JNK signalling leads to decreased insulin secretion via nucleocytoplasmic translocation of PDX-1, a key transcription factor that drives insulin expression through association with the insulin gene promoter (Figure 2) [158].

Reactive oxygen and nitrogen species production is critical to the regulation of metabolism. For instance, NO regulates the interaction between glucokinase and insulin secretory granules [159] and also affects insulin granule docking with the plasma membrane through S-nitrosylation of syntaxin 4 [160]. Generation of low- to mid-range H_2_O_2_ levels may positively regulate mitochondrial Ca^2+^ influx [161], which is critical for increasing tricarboxylic acid cycle activity and thus the second phase of insulin secretion [162]. However, low levels of physical activity and overnutrition at the whole body level cause elevated blood glucose and lipids which can promote higher generation rates of reactive oxygen and nitrogen species leading to cellular dysregulation and thus toxicity (Figure 1).

Pancreatic *β*-cells have a high metabolic activity; however, these cells are vulnerable to oxidative stress, since these cells express low levels of ROS/RNS detoxifying enzymes, such as catalase (CAT) and glutathione peroxidase (GPx) [126, 157]. Thereby, glutaredoxin and thioredoxin antioxidant reactions, which are mediated by the glutathione (GSH) system, are critical for *β*-cells [126]. GSH (L-*γ*-glutamyl-L-cysteinylglycine) can directly react with ROS or be a cofactor for GPx activity. GSH* de novo* synthesis is dependent on cysteine and especially glutamate; however, the rate-limiting step is glutamate, which is usually donated from glutamine [163]. Glutamine is the most abundant amino acid in the circulation and is considered a key metabolic mediator of insulin secretion in the presence of glucose or leucine. However, glutamine levels are decreased in T2DM [164].

Interestingly, glutamine is also an influential modulator of the Heat Shock Protein (HSP) response, which may be activated following an oxidative insult or increased endoplasmic reticulum (ER) stress. Although HSPs act as molecular chaperones for proteins damaged by oxidative processes and thus act intracellularly, they are recognised as cytoprotective agents [163, 165]. Conversely, extracellular HSP72 decreased *β*-cell insulin secretion, modified cellular bioenergetics, and initiated apoptosis* in vitro* [166]. This pathogenic extracellular release of HSP70 from tissues reacting to adverse metabolic conditions or trauma, may be common to many chronic diseases and is under current investigation.

Oxidative stress in *β*-cell may also occur through heme oxygenase-1 (HO-1), which degrades prooxidant heme into equimolar quantities of biliverdin-IX*α* (further converted to bilirubin), carbon monoxide (CO), and ferrous iron (Fe^2+^). Simultaneous production of CO and Fe^2+^ may impact *β*-cell insulin secretion [167]. Although CO gas may regulate insulin secretion via mobilisation of cAMP and cGMP, high iron concentration was associated with impaired insulin elimination from the liver and reduced insulin secretion and action [47]. Together with lipotoxicity and glucotoxicity effects, excessive ferrous iron (Fe^2+^) raises ROS/RNS through Fenton reactions. Similar effects can also be observed in the brain where CO and Fe^2+^ can modulate hippocampal synaptic activity and potentially be protective at low concentrations [168], whereas at higher levels they are neurotoxic [169]. Other biproducts of HO-1 activity within the heme oxygenase/biliverdin reductase pathway (BVR), biliverdin (BV) and bilirubin (BR), also have well known activities in scavenging ROS/RNS [170, 171]. These products from the BVR pathway are markedly increased in brain and other tissues when antioxidant systems (i.e., glutathione and catalase) are reduced (i.e., such as that observed in neurodegeneration) [172–174] and have been shown to have greater scavenging capacity for ROS/RNS than dietary antioxidants such as *α*-tocopherol in rat brain microsomes [171].

The role of HO-1 in T2DM and AD is further highlighted by the correlation between increased levels of HO-1 with brain oxidative markers [47] and links with insulin resistance and insulin signalling [175]. For example, IGF-1 administered to rats following spinal cord injury inhibits HO overexpression and CO production in neurons [176]. Treating neuronal cells with berberine, a herbal antidiabetic that improves insulin sensitivity, attenuated H_2_O_2_ induced cell toxicity, reduced ROS production, and increased antioxidant defences and HO-1 [177]. This effect was inhibited by PI3K inhibitor indicating that the benefits of barberine were dependent on PI3K/Akt signalling. A more recent study showed further evidence for a role in HO-1 in insulin resistance [178], although it did not explore subsequent effects on the brain. The findings from Jais and colleagues (2014), through several lines of evidence, suggested that HO-1 has a key role in insulin resistance and diabetes. HO-1 expression increased in liver and adipose biopsies from nondiabetic obese individuals and correlated directly with metabolic dysregulation in these individuals and was a major predictor of increased HOMA-IR levels. Similar findings were found in mice fed a high fat diet, where liver HO-1 expression increased and correlated with insulin resistance in these mice. The authors further found that hepatocyte HO-1 knockout mice were insulin hypersensitive, while overexpression of HO-1 exacerbated insulin resistance, steatosis, and metabolic dysfunction. Similar findings were reported in macrophage knockout HO-1 mice where reduced secretion of proinflammatory cytokines, blunted NF-*κ*B signalling, and reduced oxidative phosphorylation and ROS production and signalling indicated that these mice were resistant to metabolic disease. Given the links between HO-1 and insulin resistance and metabolic dysfunction, it would be interesting to determine if changes in HO-1 expression correlated with cognitive impairment or AD brain pathology or if HO-1 has roles in neuronal dysfunction. Similar studies to that of Jais and colleagues in AD mouse models may provide further insight into the role of HO-1 in AD.

### 5.2. Neurons, Oxidative Stress, and Development of AD

The high content of lipids, high requirement for oxygen, and the scarcity of antioxidant defence mechanisms make the brain highly susceptible to oxidative stress. Oxidative stress is well recognised to play a major role in the neurodegenerative process in the AD brain [179, 180]. Markers of oxidative stress, oxidised lipids, proteins, and ROS production all feature prominently in AD and other neurodegenerative diseases. Enzymes involved in metabolic pathways including glycolysis and the Krebs cycle are oxidised not only in the AD brain but also from those that have mild cognitive impairment, suggesting that these changes are an early feature of the disease process [47, 181]. As a result of these changes cerebral glucose metabolism is reduced leading to reduced ATP synthesis, contributing to disruption of neuronal functioning, loss of synapses, and overall neurodegeneration [41, 47].

As a major organelle for many biological functions including ATP synthesis and also a major site for ROS generation, it is not surprising that mitochondria dysfunction is prominent in both T2DM and AD [182]. Reductions in mitochondrial enzyme activity and oxidative stress have been shown to be early events in the disease process and in AD mouse models occur prior to amyloid plaque accumulation [183]. This may suggest that the resulting ROS generation and oxidative processes occur before any significant accumulation or A*β*. However,* in vitro* evidence suggests that the more neurotoxic A*β* oligomers can reduce cytochrome oxidase activity and increase ROS generation [184]. It remains to be determined if the oligomers are initiators of this process; however, there is evidence that age-related impairments in mitochondrial function and subsequent ROS generation are a driving force for neurodegeneration [185]. Whether this occurs prior to accumulation of A*β* (particularly oligomers) requires further evaluation in* in vivo* experiments. However, A*β*-mediated mitochondrial impairment and ROS production may induce a vicious cycle leading to further impairments in insulin signalling in AD (Figure 2). This process may be stimulated through JNK signalling pathways activated by oxidative stress, leading to insulin resistance in skeletal muscle and liver [2, 13, 65] and in the brain [186].

The oxidative processes described above as a result of insulin resistance and *β*-cell dysfunction could also have major contributions although whether there is a cause or effect relationship remains to be determined. Animal models of brain insulin resistance/deficiencies through intracrebrovascular injections of streptozotocin show abnormalities in mitochondrial function [187], which was associated with increased levels of endogenous rodent A*β*. Rodent A*β* does not aggregate into oligomers; thus the effects of A*β* accumulation independent of the effects of impaired insulin signalling could not be evaluated. More recently, injection of A*β* into the hippocampus of diabetic and nondiabetic rats resulted in metabolic disturbances in energy intake, fat oxidation, and increased carbohydrate oxidation and energy expenditure (Figure 2). These effects, however, were independent of diabetes status (i.e., diabetes did not exacerbate conditions) but are consistent with other studies that suggest that A*β* can cause metabolic dysfunction [107, 111]. Indeed, a very recent study showed that i.c.v. injection of A*β* oligomers into mice induced metabolic changes in muscle and adipose tissue consistent with insulin resistance (impaired signalling and translocation of GLUT-4) and also increased noradrenaline levels consistent with oligomers impairing peripheral sympathetic control [188]. The authors further showed the binding of Ab oligomers to dendrites of hypothalamic neurons and the subsequent increased generation of ROS and a TNF-*α*-mediated increase in eIF2*α*-P and suggest impairments in function of the metabolic/weight control centre of the brain; the hypothalamus can mediate in part the metabolic changes in the periphery. This is consistent with studies in experimental models of obesity and in humans where inflammation and neuronal injury are featured in the hypothalamus [189, 190]. More relevant animal models that perhaps mimic the clinical progression of both T2DM and AD pathologies are required to study the relationship between A*β*, mitochondrial/dysfunction/oxidative stress, and peripheral metabolic dysfunction to understand underlying molecular mechanisms.

## 6. Conclusion

T2DM and AD are age-related pathological conditions, which impact health quality. At present, there is no cure, only symptomatic treatments for these diseases. Interestingly, the methods to reduce the risk of complications associated with insulin resistance and/or diabetes also shows benefits for reducing risk of AD, for example, regular physical activity and adherence to a fat and carbohydrate controlled diet. The chronic overconsumption of foods rich in carbohydrates and various saturated lipids affects insulin secretion and has major impact on cerebral glucose metabolism. Common intracellular mechanisms in T2DM and AD include aberrant redox regulation, oxidative stress, and active inflammatory processes resulting in impaired insulin secretion and signalling. Considering the alarming worldwide numbers of people with chronic insulin resistance, diabetes, and AD, intense research is now required for identifying risk, early diagnosis, and optimal treatment for these costly, damaging, and distressing diseases.
